# Novel Immunotherapeutic Strategies for Castration-Resistant Prostate Cancer: Mechanisms and Clinical Advances

**DOI:** 10.3390/cimb48030282

**Published:** 2026-03-05

**Authors:** Xuantao Xia, Ziwei Xia, Lili Yu

**Affiliations:** 1 Faculty of Chinese Medicine, Faculty of Medicine, Macau University of Science and Technology, Macau 999078, China; 2240015369@student.must.edu.mo; 2 Guangxi Innovation Center of Zhuang Yao Medicine, Institute of Traditional Chinese and Zhuang-Yao Ethnic Medicine, Guangxi University of Chinese Medicine, Nanning 530200, China; xiaziwei2023@stu.gxtcmu.edu.cn; 3 State Key Laboratory of Mechanism and Quality of Chinese Medicine, Macau University of Science and Technology, Macao 999078, China; 4 Macau University of Science and Technology Zhuhai MUST Science and Technology Research Institute, Zhuhai 519099, China

**Keywords:** castration-resistant prostate cancer, immunotherapy, drug resistance, vaccinotherapy, combined therapy

## Abstract

Prostate cancer frequently progresses to lethal, drug-resistant castration-resistant prostate cancer (CRPC), where conventional therapies often fail due to intrinsic and acquired resistance mechanisms. This resistance creates a critical therapeutic impasse, leaving patients with limited options and poor prognoses. Immunotherapy has emerged as a promising strategy to harness the immune system against these treatment-refractory tumors, offering a potential avenue to overcome the immunosuppressive barriers that underlie CRPC drug resistance. This review synthesizes findings from a structured search of PubMed, Web of Science, and Embase (2020–2025), revealing significant clinical progress: 4 vaccine trials, 5 immune checkpoint inhibitor trials, 18 combination therapy trials (≥2 agents), and 6 targeted drug trials have been conducted. Preliminary efficacy was observed in novel approaches like bispecific antibodies (e.g., Xaluritamig achieving 59% PSA50 response), PSMA-CAR-T (P-PSMA-101), and oncolytic viruses (Ad5 PSA/MUC-1/brachyury). Basic research identified four targeted resistance mechanisms (e.g., AR-LLT1, Pygo2, and HnRNP L) and one nanoparticle-mediated triple-combination therapy (CM-AMS@AD NPs integrating photothermal, chemotherapy, and immunotherapy), which enhanced cytotoxic T-cell infiltration and suppressed CRPC growth preclinically. These collective findings suggest the potential of immunotherapy for CRPC in overcoming resistance barriers and improving patient outcomes, with bispecific T cell engagers (Xaluritamig, 59% PSA50) and PSMA-directed CAR-T therapy (P-PSMA-101, >50% PSA reduction) emerging as the most promising near-term candidates and biomarker-stratified combinations (nivolumab plus rucaparib, 84.6% PSA50, in HRR-deficient patients) illustrating the transformative power of precision patient selection; however, these findings require validation in larger, biomarker-stratified trials before definitive conclusions can be drawn. Translating this potential into clinical reality requires optimized patient selection through predictive biomarkers and rigorously validated Phase III trials to confirm durable clinical responses and long-term survival benefits.

## 1. Introduction

Castration-resistant prostate cancer (CRPC) marks a critical, often fatal stage of progression where tumors develop resistance to initially effective therapies. While standard androgen-deprivation therapy (ADT) [1], chemotherapy (e.g., taxanes) [2], and next-generation androgen receptor (AR) inhibitors (e.g., enzalutamide and abiraterone) [3,4] demonstrate initial clinical benefit, therapeutic efficacy diminishes over time as tumors evolve escape mechanisms. Ultimately, virtually all patients progress to CRPC through androgen-dependent adaptations (AR splice variants [5] or AR overexpression [3]) or androgen-independent pathways (DNA repair defects [6], PI3K/AKT/mTOR [7], or neuroendocrine differentiation) [8]. This universal resistance has created an urgent therapeutic impasse, with metastatic CRPC (mCRPC) remaining the fifth leading cause of cancer death globally [9]. Immunotherapy has emerged as a promising strategy to harness the immune system against treatment-refractory tumors. However, prostate cancer’s immunologically “cold” phenotype—characterized by a low tumor mutational burden, sparse cytotoxic T-cell infiltration [10], and an immunosuppressive microenvironment—has historically limited the efficacy of monotherapies like immune checkpoint inhibitors (ICIs) [11].

Over the past five years (2020–2025), significant progress has been made across clinical and preclinical domains. Clinically, combination strategies have demonstrated enhanced efficacy: ICIs paired with chemotherapy (e.g., pembrolizumab + docetaxel in KEYNOTE-921; median OS: 19.6 months) [12], radiotherapy (e.g., avelumab + stereotactic ablative radiotherapy; rPFS: 8.4 months) [13], or targeted agents (e.g., nivolumab + rucaparib; rPFS: 8.1 months) remodel the tumor microenvironment and improve outcomes [14]. Novel immunotherapies have also shown promise, including bispecific T-cell engagers (e.g., Xaluritamig targeting STEAP1 × CD^3^ [15]; 59% PSA50 response), PSMA-directed CAR-T cells (P-PSMA-101; >50% PSA reduction) [16], and oncolytic vaccines (e.g., Ad5 PSA/MUC-1/brachyury) [17]. Concurrently, basic research has elucidated resistance mechanisms such as docetaxel-induced suppression of natural killer (NK) cell cytotoxicity via the AR-LLT1 axis [18] and identified key mediators of immune evasion (e.g., chromatin remodeler Pygo2 [19] and splicing factor HnRNP L) [20]. Innovative approaches like multifunctional nanoparticles (CM-AMS@AD), integrating photothermal therapy, chemotherapy (DTX), and immunotherapy (Alisertib), have demonstrated potent antitumor activity in preclinical CRPC models by enhancing T-cell infiltration and dendritic cell maturation [21].

While several reviews have addressed immunotherapy in prostate cancer, the existing literature has notable limitations: earlier reviews (pre-2022) predate the availability of pivotal data from KEYNOTE-921 and Xaluritamig Phase I trials; more recent summaries tend to focus narrowly on either clinical trials or mechanistic research, but rarely integrate both within a unified analytical framework; and no prior review has systematically compared benefit–risk profiles across the full spectrum of immunotherapeutic modalities—vaccines, checkpoint inhibitors, combination regimens, and next-generation targeted agents—within a single 2020–2025 time window. The present review addresses these gaps in three specific ways. First, it provides the most comprehensive synthesis to date of completed immunotherapy trials for CRPC from 2020 to 2025, covering 33 clinical trials across four therapeutic categories, with explicit comparison of efficacy gradients, toxicity profiles, and benefit–risk trade-offs. Second, it uniquely integrates clinical trial findings with concurrent basic research advances in resistance biology (AR-LLT1, Pygo2, and HnRNP L) and therapeutic engineering (CM-AMS@AD nanoparticles), illustrating the mechanistic rationale driving next-generation combination strategies. Third, it moves beyond descriptive enumeration to provide evidence-based, stratified recommendations for therapeutic prioritization—identifying Xaluritamig and P-PSMA-101 as the highest-priority near-term candidates and biomarker-stratified combination therapy as the most clinically validated approach—thereby serving to clarify which directions warrant accelerated clinical investment.

### Search Strategy

A structured literature search was conducted across PubMed/MEDLINE, Web of Science, and Embase, supplemented by manual screening of ClinicalTrials.gov for registered trials. The search covered publications from January 2020 to December 2025.

Search terms were applied in Boolean combination: (“castration-resistant prostate cancer” OR “CRPC” OR “mCRPC”) AND (“immunotherapy” OR “immune checkpoint inhibitor” OR “PD-1” OR “PD-L1” OR “CTLA-4” OR “CAR-T” OR “bispecific antibody” OR “cancer vaccine” OR “combination therapy” OR “targeted therapy”).

Inclusion criteria: (1) clinical trials (Phase I–III) or preclinical studies reporting immunotherapeutic interventions for CRPC or mCRPC; (2) studies reporting at least one quantitative efficacy or mechanistic outcome; (3) English-language, peer-reviewed publications from January 2020 to December 2025.

Exclusion criteria: (1) studies on non-immunotherapeutic interventions without an immunotherapy component; (2) case reports, editorials, letters, or conference abstracts without full data; (3) duplicate publications reporting the same trial cohort.

Following screening and duplicate removal, 33 clinical trials and 4 preclinical studies were included in the final synthesis.

## 2. Clinical Trial Progress

The period 2020–2025 witnessed significant clinical investigation into diverse immunotherapeutic strategies for metastatic castration-resistant prostate cancer (mCRPC). Completed trials explored a broad spectrum of approaches, encompassing vaccine therapy, immune checkpoint inhibitors (ICIs), combination regimens integrating immunotherapies with other modalities, and novel targeted immunotherapies. While monotherapy with ICIs generally demonstrated limited efficacy in unselected mCRPC populations, several combination strategies and next-generation agents showed promising signals of activity, highlighting the evolving landscape of immunotherapy in this disease.

Sipuleucel-T is an active cellular immunotherapy intended to induce a T-cell immune response. To date, it remains the only approved cancer vaccine for asymptomatic or minimally symptomatic patients with metastatic castration-resistant prostate cancer (mCRPC). This therapy consists of autologous peripheral blood mononuclear cells (PBMCs), which are activated ex vivo with a recombinant fusion protein (PA2024) comprising prostate antigen, acid phosphatase, and granulocyte–macrophage colony-stimulating factor (GM-CSF), thereby promoting the expansion of antigen-loaded antigen-presenting cells (APCs). Following 36–44 h of in vitro culture, these cells are reinfused into the patient to sensitize and activate autologous T cells [22]. Relative to placebo, Sipuleucel-T prolonged median overall survival by 4.1–4.5 months [22,23] and reduced the risk of death by 22–33% [24]. In addition, preliminary safety and efficacy data for multiple therapeutic strategies in mCRPC, including vaccines, immune checkpoint inhibitors, combination regimens, and receptor signaling inhibitors, are still emerging. The clinical trial results from 2020 to 2025 are summarized below. (PSA: prostate-specific antigen; EFS: event-free survival; PFS: progression-free survival; OS: overall survival.)

### 2.1. Vaccinotherapy

Vaccinotherapy harnesses the immune system to target tumor-specific antigens, aiming to induce durable antitumor responses in castration-resistant prostate cancer (CRPC). Recent advances include viral vectors, peptide-based vaccines, and combinatorial approaches with immune checkpoint inhibitors (ICIs). These strategies seek to overcome immune tolerance while minimizing toxicity. Table 1 summarizes completed vaccine clinical trials (2020–2025), highlighting antigen targets, safety profiles, and efficacy outcomes.

### 2.2. Immunotherapy with Checkpoint Inhibitors

Immune checkpoint inhibitors (ICIs) restore T-cell-mediated antitumor activity in CRPC by blocking inhibitory signaling pathways, such as PD-1/PD-L1. While monotherapy has shown limited efficacy, biomarkers such as PD-L1 expression and DNA repair defects are being explored to identify responsive subgroups. Current trials focus on optimizing dosing and patient selection. Table 2 details completed ICI trials (2020–2025), including safety data and clinical response rates.

Collectively, the ICI trials summarized in Table 2 reveal a consistent and clinically important pattern: monotherapy PSA50 response rates remain below 11% across unselected mCRPC populations (nivolumab: 10.5%; atezolizumab: 8.6%; pembrolizumab: <10%), reflecting the immunologically “cold” phenotype of CRPC—characterized by a low tumor mutational burden, sparse CD8^+^ T cell infiltration, and an immunosuppressive tumor microenvironment dominated by myeloid-derived suppressor cells and regulatory T cells. In addition to this “cold” phenotype, prostate cancer-specific stromal and metabolic immunosuppressive mechanisms may further constrain ICI efficacy [31,32]. Prostatic acid phosphatase (PAP), particularly its transmembrane form (TMPAP), may contribute to extracellular adenosine generation and downstream immunosuppressive signaling, thereby attenuating antitumor T-cell activity [31,33]. The Phase III atezolizumab plus enzalutamide trial (NCT03016312) represents the most instructive failure: despite a mechanistically sound rationale for combining androgen receptor blockade with PD-L1 inhibition, the combination produced an inferior OS compared with enzalutamide alone (15.2 vs. 16.6 months) while it introduced treatment-related grade 5 adverse events in 1.9% of patients—a clearly unfavorable benefit–risk profile. Reactive/desmoplastic stroma, including CAF-ECM remodeling and stromal immunoregulatory signaling (e.g., TGF-β-associated programs), may create molecular and physical barriers to T-cell trafficking/infiltration and promote immune exclusion, which may partially explain the limited efficacy of checkpoint blockade in unselected CRPC populations [34,35]. This outcome underscores that overcoming CRPC’s immune evasion requires more than simply adding an ICI to hormonal therapy; rather, it necessitates biomarker-stratified patient selection (e.g., PD-L1 expression, DNA repair defects, and TMB) and mechanism-informed combination strategies to identify the minority of patients with immunologically responsive tumors [31,32].

### 2.3. Combined Therapy

Combination strategies integrate immunotherapies with chemotherapy, targeted agents, radiotherapy, or hormonal therapies to enhance synergistic effects and overcome resistance in CRPC. Approaches include ICIs with androgen receptor inhibitors, chemotherapy, PARP inhibitors, or vaccine platforms to amplify immune activation and prolong survival. Table 3 outlines key completed combination trials (2020–2025), emphasizing tolerability and survival outcomes.

Across the combination regimens in Table 3, a meaningful efficacy gradient is discernible. Chemotherapy-based combinations produced the most clinically validated survival data: pembrolizumab plus docetaxel (KEYNOTE-921) achieved a median OS of 19.6 months in a Phase III population, and ADXS31-142 plus pembrolizumab yielded a median OS of 33.7 months in a Phase I/II setting—the longest OS observed among all combination regimens reviewed. In contrast, ICI-ICI combinations (nivolumab plus ipilimumab, CheckMate 650/STARVE-PC) consistently failed to meet primary endpoints, despite high-grade toxicity in 42–53% of patients; the likely explanation is that dual checkpoint blockade cannot overcome CRPC’s fundamentally immunosuppressed microenvironment in unselected patients, and the substantial toxicity burden (four treatment-related deaths in CheckMate 650) renders this approach non-viable without prior biomarker enrichment. A critical toxicity–efficacy trade-off is also evident in the cabozantinib plus atezolizumab cohort (NCT03170960): grade 3–4 adverse events occurred in 55% of patients, yet the objective response rate was only 23%—a modest benefit that must be weighed carefully against the substantial tolerability burden. By contrast, nivolumab plus rucaparib demonstrated a favorable benefit–risk profile specifically in patients with homologous recombination repair (HRR) defects, achieving a PSA50 response rate of 84.6% in the HRR-deficient subgroup, illustrating that biomarker selection fundamentally transforms the therapeutic calculus.

### 2.4. Targeted Drug Therapy

Novel targeted agents exploit specific molecular features of CRPC, such as PSMA, STEAP1, or CD46 expression, using bispecific antibodies, antibody-drug conjugates (ADCs), and CAR-T cells. These therapies aim to improve tumor specificity while reducing off-target effects. Recent innovations include non-viral CAR-T platforms and T-cell engagers. Table 4 catalogs completed targeted therapy trials (2020–2025), focusing on mechanisms, safety, and response rates.

## 3. The Basic Research Progress of Immunotherapy for Castration-Resistant Prostate Cancer

In recent years, significant strides have been made in understanding the fundamental mechanisms underpinning immunotherapy for castration-resistant prostate cancer (CRPC). Basic research has yielded crucial mechanistic insights into both enhancing intrinsic immune cell activity and overcoming tumor resistance pathways. Key advances include the identification of novel molecular targets within CRPC cells (such as AR-LLT1 signaling [18], PYGO2 [19], and hnRNP L [20]) that modulate interactions with effector immune cells like NK cells and cytotoxic T lymphocytes (CTLs) [19], as well as the development of innovative combinatorial platforms integrating immunotherapy with other treatment modalities. This section highlights foundational discoveries elucidating how targeted disruption of specific oncogenic signals can sensitize CRPC to immune attack and explores the promising preclinical development of multifunctional therapeutic strategies designed to synergistically activate the immune system against CRPC.

### 3.1. Targeted Therapy

**Figure 1:**
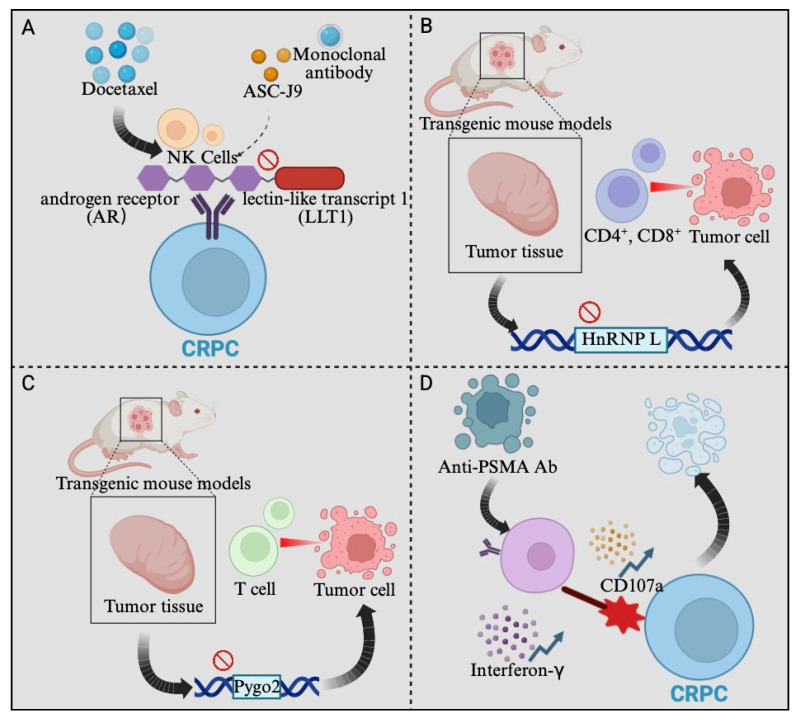
(**A**) Targeting AR with ASC-J9 or blocking LL1 by anti-human LLT1 monoclonal antibody. (**B**) Knocking out HnRNP L inhibits the development of tumors. (**C**) Knocking out Pygo2 inhibits the development of tumors. (**D**) Anti-PSMA antibody enhances NK cell-mediated cytotoxicity against CRPC cells, evidenced by upregulation of CD107a and increased interferon-γ secretion. Created in BioRender. Xia, X. (2026). BioRender.com/42tex64 (accessed on 10 January 2026).

Recent studies have shown that docetaxel may suppress the efficacy of NK cell-mediated immunotherapy against CRPC cells in vitro through androgen receptor (AR)-lectin-like transcript 1 (LLT1) signaling. Mechanistic analysis revealed that docetaxel acts by increasing AR expression, thereby upregulating LLT1 in CRPC cells, ultimately suppressing NK cell-mediated antitumor immunity (Figure 1A) [18]. In addition, Pygopus 2 (PYGO2) has been identified as a driver oncogene within the 1q21.3 amplicon in prostate cancer. Using transgenic mouse models of metastatic prostate adenocarcinoma, researchers found that PYGO2 deletion slowed tumor growth, reduced metastases, and prolonged survival. PYGO2 loss enhanced cytotoxic T lymphocyte (CTL) activation/infiltration and tumor cell sensitivity to T cells. Mechanistically, PYGO2 orchestrates a p53/Sp1/Kit/Ido1 network, creating an immunosuppressive niche. Inhibiting PYGO2 (genetically or pharmacologically) boosted immunotherapy efficacy (ICB, adoptive transfer, and MDSC targeting). Clinically, high PYGO2 correlates with reduced CD8^+^ T cells and worse ICB outcomes. Targeting PYGO2 may improve immunotherapy for advanced prostate cancer (Figure 1C) [19]; HnRNP L deficiency enhances CD4^+^/CD8^+^ T cell infiltration and inhibits tumorigenesis. Mechanistically, HnRNP L boosts c-Myc translation via EIF4G1 alternative splicing, increasing CXCL8 secretion. In vivo, the EIF4G1 inhibitor SBI-0640756 attenuated HnRNP L-driven tumor progression and immunosuppression. Critically, HnRNP L knockdown synergized with anti-PD-1 therapy to suppress prostate cancer xenograft growth (Figure 1B). This reveals HnRNP L’s role in immune regulation and offers a strategy to enhance CRPC immunotherapy [20]. In a castration-resistant prostate cancer (CRPC) model using Myc-CaP cells in immunocompetent mice, granulocytic myeloid-derived suppressor cells (G-MDSCs) increased significantly within the tumor microenvironment during disease progression. Screening multiple immunotherapies revealed that interferon-α (IFNα) was more effective than anti-PD-L1, anti-CTLA-4, anti-4-1BB, IL-2, or IL-9 in suppressing tumor growth. IFNα reduced both the quantity and immunosuppressive function of granulocytic myeloid-derived suppressor cells (G-MDSCs) in the tumor microenvironment (TME)—inhibiting their differentiation in vitro, decreasing their numbers in vivo, and diminishing their suppression of T cell proliferation and activation. These findings establish G-MDSCs as critical immunotherapy targets in CRPC and position IFNα therapy as a promising therapeutic strategy due to its ability to counteract G-MDSC-mediated immunosuppression and restore T cell function [56]. Critically, this G-MDSC-driven immunosuppressive niche provides a mechanistic explanation for the consistent failure of ICI monotherapy in unselected CRPC populations (Table 2): anti-PD-L1 and anti-CTLA-4 agents act on T cell checkpoint pathways but do not address the upstream myeloid barrier that actively depletes and exhausts tumor-infiltrating lymphocytes. The superior efficacy of IFNα over anti-PD-L1, anti-CTLA-4, and other immunotherapies in the Myc-CaP model is therefore not merely a preclinical observation, but a mechanistic argument for why ICI monotherapy is insufficient and why combination strategies targeting both checkpoint inhibition and G-MDSC depletion may be required to achieve durable responses in CRPC. Interferon-γ (IFNγ) consistently upregulated MHC-I (in vitro/vivo) and PD-L1 (in vitro) across diverse prostate cancer subtypes, while reducing E-cadherin expression. This E-cadherin decrease enhanced apoptosis when IFNγ-pretreated mCRPC cells received chemotherapy combined with a death cytokine [57]. The study investigated the USP10/METTL3/CXCR4 axis in CRPC immunotherapy. CXCR4 was upregulated in CRPC tissues/cells, and its knockdown inhibited tumor cell proliferation, migration, and invasion; reduced M2 macrophage polarization/recruitment; and mitigated CD8^+^ T cell exhaustion. CXCR4 activated the JAK2/STAT3 pathway to promote CCL2 and PD-L1 expression. Furthermore, USP10 regulated CXCR4 expression via METTL3. Pharmacological JAK2 inhibition (AG490) blocked CXCR4-driven oncogenic effects. These findings establish the USP10/METTL3/CXCR4 axis as a significant regulator of the CRPC immune microenvironment and highlight CXCR4 as a promising therapeutic target [58].

### 3.2. Combination Therapy

**Figure 2:**
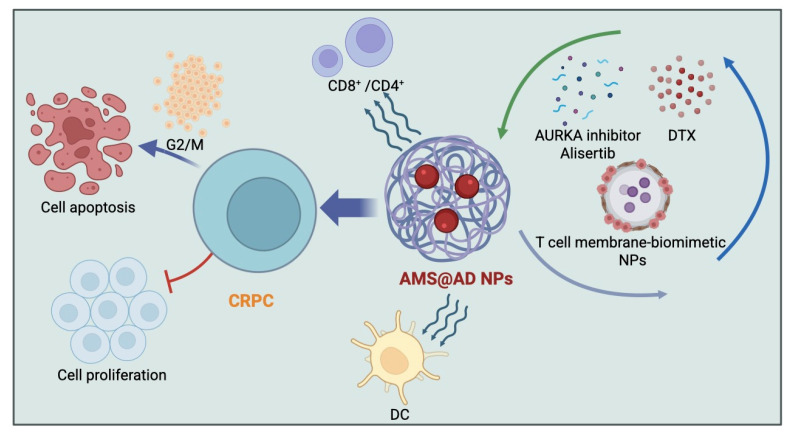
The T cell membrane-encapsulated CM-AMS@AD nanoparticles (loaded with Alisertib and DTX) significantly inhibit CRPC growth and induce apoptosis through a triple-combination approach integrating photothermal therapy, chemotherapy, and immunotherapy, mediated via CD8^+^/CD4^+^ T cell activation, dendritic cell stimulation, G2/M cell cycle arrest, and induction of cell apoptosis, while suppressing cell proliferation. Created in BioRender. Xia, X. (2026). BioRender.com/cdk3rwv (accessed on 10 January 2026).

Researchers developed an anti-PSMA antibody by immunizing a llama with human PSMA protein, isolating anti-PSMA camelid heavy-chain antibody variable domain (VHH) clones via phage display, and recombinantly fusing the VHH to a human Fc region. This anti-PSMA Ab significantly enhanced NK cell-mediated cytotoxicity against CRPC cells in vitro, as evidenced by an increased killing rate, upregulation of CD107a, increased interferon-γ secretion, and decreased PSA levels (Figure 1D). The study also demonstrated strong antitumor effects in patient-derived organoid (PDO) and CRPC xenograft mouse models. Combined treatment with anti-PSMA Ab and a high dose of human peripheral blood-derived natural killer (PB-NK) cells improved antitumor efficacy against CRPC and was proposed as a promising clinical strategy for CRPC [57]. Targeting Aurora-A kinase (AURKA) has also shown promise in cancer therapy. One study evaluated ART-T cell membrane-encapsulated AMS@AD (CM-AMS@AD) nanoparticles (NPs) in a photothermal–chemotherapy–immunotherapy combination strategy for CRPC. Bioinformatic analysis of the TCGA-PRAD dataset showed AURKA overexpression in PCa, which was associated with poor clinical outcomes, while single-cell RNA sequencing data from the GEO database showed a significant reduction in immune cells in CRPC. T cell membrane-biomimetic NPs loaded with the AURKA inhibitor Alisertib and DTX were synthesized and characterized by dynamic light scattering and transmission electron microscopy, showing good stability and uniformity (average diameter, 158 nm). In vitro, these NPs inhibited CRPC cell proliferation, increased the G2/M cell population, and promoted apoptosis, as confirmed by γH2AX expression. In vivo, CM-AMS@AD NPs accumulated in tumor tissues, significantly slowed tumor growth, reduced proliferation, increased apoptosis, and improved the immune microenvironment by promoting dendritic cell (DC) maturation and increasing CD8^+^/CD4^+^ ratios. These findings suggest that CM-AMS@AD NPs may represent a promising triple-combination strategy for CRPC, integrating photothermal therapy, chemotherapy, and immunotherapy, with potential for future clinical application (Figure 2) [21]. However, clinical translation will require addressing the CMC challenges of this multi-component architecture—including batch-to-batch consistency of membrane coating and dual drug encapsulation—as well as systematic evaluation of potential cumulative toxicity from three simultaneously delivered modalities, which may be underestimated in murine models. Combining enzalutamide and Enhancer of zeste homolog 2 (EZH2) inhibitor GSK-126 synergistically inhibited CRPC growth and neuroendocrine differentiation in mouse models. This antitumor effect required an intact immune system, as activity was lost in immunodeficient mice. The combination enhanced tumor-specific CD8^+^ T cell cytotoxicity and IFN-γ production. These results support using this combination for CRPC and suggest immunotherapy potential [59]. A pre-modification strategy engineered dendritic cells to produce extracellular vesicles highly expressing XCL1 (DEXXCL1), alongside CCR7 and MHC I. In vitro and in vivo models demonstrated that these DEXXCL1 vesicles enhance dendritic cell uptake, recruit and activate cDC1 cells within tumors, and boost antigen presentation. Combined with cisplatin-induced immunogenic cell death (releasing STEAP1 antigens), this nanovaccine strategy significantly increased CD8^+^ T cell proliferation and cytotoxicity while reducing regulatory T cells and immunosuppressive factors, effectively reshaping the tumor microenvironment. This synergy potently inhibited prostate tumor growth and prolonged survival in mouse models [60]. Previous studies have discovered a combinatorial strategy for metastatic castration-resistant prostate cancer (mCRPC) using γδ-enriched CAR-T cells targeting PSCA and zoledronate (ZOL). In a bone mCRPC mouse model, these CAR-T cells induced rapid tumor regression, improved survival, and reduced bone disease [61]. We developed a novel approach combining enzalutamide with the EZH2 inhibitor GSK-126 to overcome enzalutamide resistance and enhance antitumor immunity in CRPC. Testing in vitro and in mouse models (subcutaneous and spontaneous) showed that the drugs synergistically reduced tumor growth and prevented neuroendocrine differentiation. This antitumor effect was ineffective in immunodeficient mice, confirming immune dependence. The combination activated tumor-specific CD8^+^ T cells, boosting their cytotoxic activity and IFN-γ production [59].

## 4. Discussion

This comprehensive review synthesizes significant advancements in immunotherapy for castration-resistant prostate cancer (CRPC) from 2020 to 2025. Clinical trial data demonstrate that while monotherapies like immune checkpoint inhibitors (ICIs) exhibit limited efficacy in CRPC (their objective response rates often being <10%) [11], combination strategies show substantial promise. This limited ICI monotherapy efficacy is mechanistically grounded in the G-MDSC-dominated immunosuppressive microenvironment identified in basic research (Section 3.1): PD-1/PD-L1 blockade cannot overcome the upstream myeloid barrier constituted by G-MDSCs, which actively suppress T cell proliferation and activation regardless of checkpoint status. This explains why the most instructive clinical failure—the Phase III atezolizumab plus enzalutamide trial—produced inferior OS despite a mechanistically sound rationale, and it underscores that effective CRPC immunotherapy must simultaneously address both T cell checkpoint exhaustion and myeloid-driven immune exclusion. Key successes include pembrolizumab plus docetaxel (median OS: 19.6 months in KEYNOTE-921) [12], nivolumab plus rucaparib (median rPFS: 8.1 months) [14], and radiotherapy combined with immunotherapies like avelumab or Sipuleucel-T [62]. Novel agents, particularly bispecific antibodies (e.g., Xaluritamig achieving 59% PSA50 response) [15], PSMA-directed CAR-T cells (P-PSMA-101 with >50% PSA reduction) [16], and oncolytic viruses (Ad5 PSA/MUC-1/brachyury), also show encouraging preliminary activity. It should be noted, however, that PSA50 response is a surrogate endpoint and does not invariably predict overall survival benefit in CRPC immunotherapy—as exemplified by Sipuleucel-T, which demonstrated a 4.1-month OS improvement in the IMPACT trial despite minimal PSA changes. Moreover, immune-mediated pseudo-progression, in which transient PSA elevations occur in the context of genuine antitumor activity, may confound PSA-based response classification for CAR-T and bispecific antibody therapies. Confirmation of durable OS benefit in adequately powered Phase II/III trials therefore remains essential before these agents can be considered definitively promising. Basic research identified crucial resistance mechanisms (e.g., docetaxel-induced NK suppression via AR-LLT1 [18] and Pygo2/HnRNP L-mediated T-cell exclusion [19,20]) and developed innovative solutions like the multifunctional nanoparticle CM-AMS@AD [21], which integrates photothermal therapy, chemotherapy (DTX), and immunotherapy (Alisertib), significantly suppressing CRPC growth preclinically by enhancing T-cell infiltration and dendritic cell maturation.

This review has several limitations that warrant acknowledgment. First, our reliance on PubMed-indexed studies may have led to the omission of relevant data from other databases (e.g., Sci-Hub and Web of Science) or ongoing trials (e.g., ClinicalTrials.gov), potentially introducing selection bias. While a structured search strategy with explicit inclusion/exclusion criteria has been documented (Search Strategy Subsection), the absence of formal risk of bias assessment across heterogeneous trials limits the potential for quantitative synthesis (e.g., meta-analysis). Future efforts should adopt the full PRISMA-guided systematic review methodology to enable more rigorous evidence synthesis. Second, while clinical outcomes (PSA response, PFS, and OS) and safety profiles were summarized, deeper critical appraisal of trial failures (e.g., CheckMate 650 and STARVE-PC) and comparative toxicity analyses across regimens were not undertaken. Additionally, several trials reviewed—particularly early-phase targeted therapy trials—relied primarily on PSA50 response as the efficacy endpoint. Given the well-documented dissociation between PSA response and OS in CRPC immunotherapy (as illustrated by the Sipuleucel-T experience) and the potential for pseudo-progression to confound PSA-based assessment, conclusions regarding clinical benefit derived from PSA endpoints alone should be interpreted with caution. Third, biomarker-driven insights remained underdeveloped: predictive biomarkers (e.g., PD-L1, AR-V7, and TMB) and tumor microenvironment features (e.g., spatial immune cell distribution) were not systematically evaluated for their utility in patient stratification. Fourth, translational gaps between preclinical findings (e.g., Pygo2/HnRNP L knockout [19] and nanoparticle therapies) and clinical applicability were inadequately addressed. In particular, the CM-AMS@AD platform faces substantial CMC manufacturing challenges and potential cumulative toxicity risks from its triple-modality design that are not fully capturable in mouse models and must be evaluated in IND-enabling studies prior to clinical translation. Finally, geographical bias toward trials from the USA, Europe, and Japan overlooks potential ethnic/genetic influences on treatment response. Future efforts should adopt PRISMA-guided systematic reviews, integrate multi-omics biomarkers for patient selection, prioritize global trial inclusivity, and rigorously evaluate real-world feasibility of novel approaches (e.g., CAR-T [16] and triple-combination nanotherapies) to bridge these gaps.

Synthesizing the clinical and preclinical evidence reviewed here, a stratified picture of therapeutic promise emerges. Among novel targeted agents, Xaluritamig (STEAP1 × CD3 bispecific antibody) and P-PSMA-101 (PSMA-directed CAR-T) represent the highest-priority candidates for near-term development: their PSA50 response rates of 59% and >50%, respectively, are substantially higher than any ICI monotherapy or combination regimen tested in unselected mCRPC populations, and both demonstrate manageable safety profiles with appropriate risk mitigation (e.g., CRS prophylaxis for Xaluritamig). Importantly, however, PSA50 response should be interpreted as a signal of biological activity rather than a confirmed surrogate for OS benefit; in CRPC immunotherapy, PSA response and overall survival can be dissociated, as Sipuleucel-T’s clinical experience illustrates. Definitive evaluation of these agents must therefore prioritize OS and radiographic progression-free survival as co-primary endpoints in future Phase II/III trials. These agents warrant accelerated progression to biomarker-selected Phase II/III trials. Among combination strategies, nivolumab plus rucaparib stands out as the clearest example of precision-guided combination therapy: the PSA50 response rate increased from 27.3% in unselected patients to 84.6% in the HRR-deficient subgroup, demonstrating that biomarker stratification—not simply adding a second agent—is the mechanism of benefit. Pembrolizumab plus docetaxel (KEYNOTE-921; median OS, 19.6 months) remains the most clinically validated combination option for unselected patients and should be considered the current standard-of-care benchmark for future trials. Conversely, dual ICI combinations (nivolumab plus ipilimumab) and non-biomarker-selected ICI plus hormonal therapy (atezolizumab plus enzalutamide) have demonstrated unfavorable benefit–risk profiles and should not be advanced without robust predictive biomarker strategies. The CM-AMS@AD nanoparticle platform [15], while highly innovative in its triple-combination design, requires clinical translation data before firm development recommendations can be made. Realizing the full potential of CRPC immunotherapy will ultimately depend on three converging priorities: biomarker-stratified trial designs to identify responsive patient subsets, mechanistically informed combination strategies that address CRPC’s immunosuppressive microenvironment, and rigorously conducted Phase III trials to confirm durable survival benefits.

## Figures and Tables

**Table 1:** Completed vaccine clinical trials for CRPC (2020–2025).

Clinical Trial Number	Trial Phase	Target	Description	Adverse Reactions	Results	Country
VANCE (NCT02390063) [22]	I	5T4-specific T-cell	A novel vaccination based on two replication-deficient viruses, chimpanzee adenovirus (ChAd) and MVA (Modified Vaccinia Ankara).	Among the common systemic AEs usually seen with post-replication-incompetent viral-vectored vaccines, feverishness, myalgia and fatigue were the most frequently reported, affecting 59%, 70% and 72% of individuals, respectively, and usually were resolved within 7 days post-vaccination.	PSA, >100% increase in PSA levels post-vaccination for 3 participants, with others showing <50% increase.	UK
KRM-20 (UMIN000011028) [23]	II	Cytotoxic T lymphocytes (CTLs) against twelve different tumor-associated antigens (TAAs) highly expressed in prostate cancer tissues	KRM-20 is a peptide vaccine composed of 20 peptides and is designed to induce cytotoxic T-lymphocyte responses against 12 tumor-associated antigens.	The most common adverse events (AEs), occurring in >40% of patients in one or both study arms, included injection-site reactions, alopecia, neutropenia, and peripheral neuropathy.	No significant difference was observed in PSA response; however, both human leukocyte antigen (HLA)-IgG and CTL responses were increased in the KRM-20 arm.	Japan
PPV(UMIN000011308) [24]	III	HLA	A personalized peptide vaccination.	Common adverse reactions include loss of appetite, fever, anemia, nausea, constipation, etc.	Median OS was 16.1 months (95% CI, 13.0–18.2 months), and median PFS was 4.2 months (95% CI, 4.0–5.6 months).	Japan
PF-06753512(PrCa VBIR) (NCT02616185) [25]	1	PSA	PrCa VBIR integrates a vaccine with ICIs and applies novel delivery strategies, including electroporation of plasmid DNA (pDNA)-encoded antigens and subcutaneous (SC) administration of ICIs.	The most common adverse events were fatigue (40.7%), influenza-like illness (30.8%), diarrhea (23.1%), immune-related thyroid dysfunction (19.8%), and rash (15.4%).	The median duration of PSA response was 3.9 (1.9–4.2) and 10.1 (6.9–28.8) months for different doses.	USA
aHyC (EUDRACT: 2012-005498-29) [26]	1/2	NK cells	Generated by electrofusion of autologous dendritic cells (DCs) and tumor cells (TCs).	The most frequent adverse event (AE) was asthenia (5 patients, 25%), followed by urinary frequency/urgency (4 patients, 20%) and urinary retention (4 patients, 20%).	The median OS was 58.5 months (95% confidence interval [CI], 38.8–78.2 months). The baseline median prostate-specific antigen (PSA) value was higher in the aHyC group (8.9 ng/mL; interquartile range [IQR], 5.6–23.7 ng/mL).	Slovenia

**Table 2:** Completed immune checkpoint inhibitor clinical trials for CRPC (2020–2025).

Clinical Trial Number	Trial Phase	Target	Description	Adverse Reactions	Results	Country
Nivolumab (NCT03040791) [27]	II	PD-L1	A PD-1 inhibitor.	Grade ≥ 3 adverse events were reported in 47.3% of participants.	The overall PSA50 response rate was 10.5% (4/38). There were no statistically significant differences in median PSA-PFS (1.9 vs. 2.8 months, *p* = 0.52) or rPFS (3.4 vs. 5.5 months, *p* = 0.70) between patients with and without DRD (DNA repair defects).	Brazil
Atezolizumab (NCT01375842) [28]	I	PD-L1	An anti-programmed death-ligand 1 (anti-PD-L1).	Treatment-related adverse events occurred in 21 patients (60.0%), with no deaths.	PSA, 8.6% response; OS, 14.7 months; overall limited efficacy, so combination approach may be needed.	USA
Pembrolizumab (NCT02787005) [29]	II	PD-L1	Monoclonal antibody pembrolizumab (PD-1 inhibitor) with previous treatment using docetaxel or enzalutamide.	Treatment-related adverse events occurred in 60% of patients, were of grade 3 to 5 severity in 15%, and led to discontinuation of treatment in 5%.	PSA, <10% response; ORR, <5%; rPFS, 2.1, 2.1, and 3.7 months for 3 cohorts. (Cohort 1: PD-L1-positive; Cohort 2: PD-L1-negative; Cohort 3: bone-predominant disease, regardless of PD-L1 expression.)	USA
ADXS31-142 + pembrolizumab (NCT02325557) [30]	I/II	PSA	ADXS31-142 is an attenuated Listeria monocytogenes-based immunotherapy targeting prostate-specific antigen (PSA).	All patients had ≥1 treatment-related adverse event, mostly grade 1/2 manageable events.	Median PFS was 2.2 months (95% CI: 0.8–7.4), median OS was 7.8 months (95% CI: 4.4–18.5).	USA
Ad5 PSA/MUC-1/brachyury (NCT03481816) [17]	I	PSA, brachyury, and MUC-1	A novel vaccine platform using adenovirus 5 (Ad5) vectors [E1-, E2b-] targeting three TAAs—prostate-specific antigen (PSA), brachyury, and MUC-1—has been developed.	The vaccine was tolerable and safe, and no grade >3 treatment-related adverse events or dose-limiting toxicities (DLTs) were observed.	Median PSA was 22 weeks (95% CI: 19.1 to 34).	USA

**Table 3:** Completed combined medication clinical trials for CRPC (2020–2025).

Clinical Trial Number	Trial Phase	Description	Adverse Reactions	Results	Country
Pembrolizumab + HER2 BAT(NCT03406858) [36]	II	HER2 bispecific antibody (HER2Bi)-armed activated T cells (HER2 BAT) and programmed death 1 inhibitor, pembrolizumab.	The toxicities were grade 1–2 infusion reactions with fever, chills, headaches, nausea and/or myalgias.	Primary endpoint of 6-month PFS was achieved in 5 out of 14 patients (38.5%; 95% confidence interval, 19.5–76.5%).	USA
Cryoablation + autologous immature DC(NCT02423928) [37]	I	Cryoablation destroys cancer tissue, releases tumor-associated antigens and creates a pro-inflammatory microenvironment, while dendritic cells (DCs) activate immune responses through processing of antigens.	No dose-limiting toxicities or grade >3 adverse events were observed among the 18 participants.	The results suggest antitumor activity, as reflected by altered T-cell receptor repertoires, and showed a 33% durable clinical benefit (>46 weeks), with a median overall survival of 40.7 months.	Norway
Avelumab + carboplatin (2017-004552-39) [38]	Ib	Avelumab: PD-L1 inhibitor.	The safety profile was considered acceptable, although grade 3–4 treatment-related adverse events occurred in 73% of patients.	A PSA response rate of ≥50% was observed in 7.7% of patients. Median radiographic PFS was 6.6 months (95% CI, 4.28–9.01 months), and median OS was 10.6 months (95% CI, 6.68–NR).	Spain
Atezolizumab +enzalutamide (NCT03016312) [39]	3	Enzalutamide, a second-generation oral androgen receptor (AR) antagonist; Atezolizumab (anti–PD-L1).	Grade 5 adverse events were reported in 4.3% of patients (16/374) receiving atezolizumab plus enzalutamide and 3.2% of patients (12/376) receiving enzalutamide alone; among these deaths, 1.9% (7/374) and 0.3% (1/376), respectively, were considered treatment-related.	Stopped early due to efficacy failure: combination arm produced inferior OS versus enzalutamide alone (15.2 vs. 16.6 months), with additional treatment-related grade 5 adverse events in 1.9% of patients—unfavorable benefit–risk profile leading to trial discontinuation.	UK
Pembrolizumab + Docetaxel (NCT03834506) [12]	III	Pembrolizumab, an anti-PD-1 antibody.	Grade ≥3 treatment-related adverse events occurred in 43.2% of participants who received pembrolizumab plus docetaxel.	At the first interim analysis (data cutoff: 27 September 2021), median rPFS was 8.6 months (95% CI, 8.3–10.2) with pembrolizumab plus docetaxel. At FA, median OS was 19.6 months (95% CI, 18.2–20.9).	USA
Avelumab + stereotactic ablative body radiotherapy (SABR) (NCT02985957) [13]	2	PD-L1 inhibitor.	Grade 3–4 treatment-related adverse events occurred in six patients (16%), and three patients (10%) required high-dose corticosteroid therapy.	Median rPFS was 8.4 mo (95% CI, 4.5—not reached [NR]), and median OS was 14.1 mo (95% CI, 8.9-NR).	Australia
ESK981 + nivolumab (NCT04159896) [40]	II	ESK981: multi-tyrosine kinase inhibitor; nivolumab: PD-1 inhibitor.	Grade 3 treatment-related adverse events (AEs) included fatigue, anemia, and lymphopenia.	The median rPFS was 3.7 months (95% CI, 1.6–8.4), and the median OS was 9.6 months (95% CI, 1.8-22.4).	USA
Pembrolizumab +enzalutamide (NCT02787005) [41]	2	Pembrolizumab: PD-1 inhibitor.	Grade ≥3 treatment-related adverse events occurred in 35 patients (27.8%).	DCR was 53.1% (95% CI, 41.7–64.3%); median overall survival was 17.6 months (95% CI, 14.0–22.6).	USA
Cabozantinib + atezolizumab (NCT03170960) [42]	1b	Cabozantinib, a tyrosine kinase inhibitor with immunomodulatory properties; atezolizumab, a PD-L1 inhibitor.	Among 132 patients, 72 (55%) experienced grade 3–4 treatment-related adverse events, with the most common being pulmonary embolism (11 [8%]), diarrhea (9 [7%]), fatigue (9 [7%]), and hypertension (9 [7%]).	Objective response rate was 23% (95% CI, 17–32; 31 out of 132 patients), with three (2%) confirmed complete responses and 28 (21%) confirmed partial responses.	USA
Atezolizumab + enzalutamide (NCT03016312) [39]	III	Atezolizumab: small-molecule atezolizumab (PD-L1 inhibitor).	Grade 5 AEs were seen in 4.3% of patients (16 out of 374).	Stopped early because patients were at risk of immune-mediated adverse events; OS, 15.2 months for atezolizumab + enzalutamide vs. 16.6 months for enzalutamide only.	UK
STARVE-PC Ipilimumab + Nivolumab (NCT02601014) [43]	II	Ipilimumab (anti-CTLA4 monoclonal antibody), nivolumab (PD1 inhibitor), some concurrent treatment with nivolumab (all with enzalutamide).	In cohort 2, sixteen grade 3–4 adverse events were reported in 8 out of 15 patients (53%).	In a subset of patients receiving immune blockade, lower alkaline phosphatase levels were observed; however, the primary endpoint was not met.	USA
Nivolumab + Ipilimumab (NCT02985957) [44]	II	Ipilimumab: CTLA4 inhibitor; nivolumab: PD1 inhibitor.	Grade 3–4 treatment-related adverse events occurred in ∼42–53% of patients, with four treatment-related deaths.	OS, 15.2 months in post-chemo cohort and 19 months in pre-chemo cohort; ORR, 10% in post-chemo cohort and 25% in pre-chemo cohort.	USA
MVI-816 + pembrolizumab (NCT02499835) [45]	II	The combination of programmed cell death 1 (PD-1) blockade and MVI-816 was safe and was able to augment tumor-specific T-cell responses.	One grade 4 event (hyperglycemia) was observed. Immune-related adverse events (irAEs) > grade 1 were reported in 42% of patients overall.	A PSA decline of >50% was observed in 10% of patients and was associated with a favorable 6-month disease control rate.	USA
ADXS31-142 + pembrolizumab (NCT02325557) [30]	I/II	ADXS31-142 is an attenuated Listeria monocytogenes-based immunotherapy targeting prostate-specific antigen (PSA).	All patients experienced at least one treatment-related adverse event, most of which were manageable grade 1/2 events. No additive toxicity was observed with the combination treatment.	Median PFS was 5.4 months (95% CI: 2.3–7.9), 33.7 months (95% CI: 15.4—not evaluable).	USA
Pembrolizumab + enzalutamide (NCT02312557) [46]	II	Pembrolizumab: PD-1 inhibitor.	Immune-related adverse events were observed in seven patients, including colitis in two patients, hypothyroidism in three, hyperthyroidism in one, and myositis in two.	Median overall survival for all patients was 21.9 months (95% CI, 14.7–28.4 months).	USA
Sipuleucel-T + Stereotactic Ablative Body Radiation (SAbR) (NCT01818986) [47]	II	Sipuleucel-T is a therapeutic cancer vaccine composed of autologous antigen-presenting cells (APCs) extracted via leukapheresis that is activated ex vivo with a fusion protein (PA2024); SAbR is a cytoreductive modality that can safely target bulky disease sites and provide immunostimulatory effects.	Treatment was well tolerated, with 51, 8, and 4 treatment-related grade 1, 2, and 3 toxicities, respectively, and no grade 4 or 5 adverse events.	Median OS was 76.8 weeks (95% CI, 41.6–130.8 weeks).	USA
Nivolumab + rucaparib (NCT03338790) [14]	2	Rucaparib: a PARP inhibitor; Nivolumab: a PD-1 inhibitor.	The most common any-grade and grade 3–4 treatment-related adverse events (TRAEs) were nausea (40.9% and 40.8%) and anemia (20.5% and 14.1%).	PSA: 27.3% (17.0–39.6) (n = 66), 41.9 (24.5–60.9) (n = 31), and 84.6% (54.6–98.1) (n = 13); median rPFS: 8.1 (5.6–10.9) (n = 71), 10.9 (6.7–12.0) (n = 34), and 10.9 (5.6–12.0) (n = 15) months; and median OS: 20.2 (14.1–22.8) (n = 71), 22.7 (14.1—not estimable) (n = 34), and 20.2 (11.1—not estimable) (n = 15) months.	France
Ipilimumab + nivolumab (NCT03570619) [48]	2	Ipilimumab: an immune checkpoint inhibitor (ICI); nivolumab: a PD-1 inhibitor.	The most common grade 1–2 AEs included fatigue (37.5%), nausea (21.9%), anorexia (15.6%), maculopapular rash (15.6%), diarrhea (12.5%), pruritis (12.5%), abdominal pain (9.4%), and arthralgias (6.3%).	The PSA50 rate was 9% (95% CI, 1–28%) with 2 responders, median PSA-PFS was 7.0 months (95% CI, 3.6–11.4), median OS was 9.0 months (95% CI, 6.2–12.3).	USA

**Table 4:** Completed targeted drug therapy clinical trials for CRPC (2020–2025).

Clinical Trial Number	Trial Phase	Target	Description	Adverse Reactions	Results	Country
Acapatamab (NCT03792841) [49]	I	Prostate-specific membrane antigen (PSMA)	A half-life extended, PSMA-targeting bispecific T-cell engager.	Cytokine release syndrome (CRS) was the most common treatment-emergent adverse event seen in 97.4% and 98.2% of patients in dose exploration and dose expansion respectively; grade ≥ 3 was seen in 23.4% and 16.1%.	Median PSA progression-free survival (PFS) was 3.3 months (95% CI, 3.0–4.9).	USA
JNJ-70218902 (JNJ-902) (NCT04397276) [50]	1	Transmembrane protein	A bispecific T-cell-redirecting antibody against TMEFF2 in metastatic castration-resistant prostate cancer.	All 82 participants experienced at least one treatment-emergent adverse event (TEAE) of any grade, which included fatigue (44 [53.7%]), injection site erythema (41 [50.0%]), decreased appetite (38 [46.3%]), anemia (30 [36.6%]), back pain (23 [28.0%]), and weight decrease (20 [24.4%]). Forty-six participants (56.1%) experienced a grade ≥3 TEAE, including anemia (14 [17.1%]) and fatigue (10 [12.2%]).	The median duration of JNJ-902 treatment across all cohorts was 1.91 months (range, 0.0–19.4).	USA
FOR46 (FG-3246) (NCT03575819) [51]	I	CD46	An immune-modulating antibody–drug conjugate targeting CD46.	The most common grade ≥3 adverse events across all dose levels were neutropenia (59%), leukopenia (27%), lymphopenia (7%), anemia (7%), and fatigue (5%).	14 out of 39 evaluable patients (36%) achieved a PSA50 response. The confirmed objective response rate was 20%.	USA
P-PSMA-101(NCT04249947) [16]	I	PSMA	It is manufactured using a novel non-viral transposon system (piggyBac). Genes encoding a PSMA-targeted Centyrin CAR, iCasp9-based safety switch, and DHFR are inserted to purify CAR-T cells.	Only 3 cases of cytokine release syndrome (CRS) were observed, all of which were of low grade (1/2).	The overall PSA reduction in the treatment group exceeded 50%.	USA
Xaluritamig (NCT04221542) [15]	1	T-cell	A STEAP1 × CD^3^ XmAb 2 + 1 immune therapy.	The most common treatment-related adverse events were cytokine release syndrome (CRS; 72%), fatigue (45%), and myalgia (34%).	Target doses ≥0.75 mg (59% PSA50; 41% ORR).	USA
JNJ-64041809(JNJ-809) (NCT02625857) [52]	1	Prostatic acid phosphatase (PAP), prostate-specific membrane antigen (PSMA), synovial sarcoma X breakpoint 2 (SSX2), and homeobox protein NKX3.1	A live-attenuated, double-deleted Listeria monocytogenes (LADD Lm)-based immunotherapy.	The most common adverse events (AEs) reported were chills (92%), pyrexia (81%), and fatigue (62%). The most frequent grade ≥3 AEs were lymphopenia (27%) and hypertension (23%).	The majority of patients had a Gleason score ≥8 (62%).	USA
JNJ-63898081 (NCT03926013) [53]	1	T-cell	A PSMA and CD3 bispecific antibody.	Serious TEAEs occurred in 18 (46.2%) patients, with the most common being CRS (20.5%), pyrexia (7.7%), and back pain (5.1%).	The best overall response observed was stable disease in 2 out of 23 participants (13.0%) who had baseline target lesions. Transient PSA decreases were observed at treatment doses greater than 30 μg/kg SC. Two patients treated with 55 μg/kg had confirmed PSA decline of >50%.	USA
PSCA-CAR T cell therapy (NCT03873805) [54]	1	CAR T cells	A chimeric antigen receptor (CAR) T cell therapy.	Two DLTs in DL2 and a maximum grade 2 CRS among the 14 participants in the study.	4 out of 14 participants showed PSA decline >30%.	USA
PSMA-targeting TGFβ-insensitive armored CAR T cells (NCT03089203) [55]	1	CAR T cells	Directed CAR T cells armored with a dominant-negative TGF-β receptor.	No treatment-related grade ≥3 AEs or cases of CRS were observed.	Median OS of 477 days (15.9 months) and PFS of 132 days (4.4 months).	USA

## Data Availability

No new data were created or analyzed in this study. Data sharing is not applicable to this article.
